# A Holistic Approach to Parasitoid–Host Interaction Along an Elevational Gradient Revealed Coevolution Driven by Host Foraging Strategy

**DOI:** 10.1002/ece3.71227

**Published:** 2025-04-11

**Authors:** Ľudmila Černecká, Radek Michalko, Jakub Sýkora, Peter Gajdoš, Pavol Purgat, Kamil Holý, Martina Dorková, Stanislav Korenko

**Affiliations:** ^1^ Institute of Forest Ecology Slovak Academy of Sciences Zvolen Slovak Republic; ^2^ Department of Forest Ecology, Faculty of Forestry and Wood Technology Mendel University in Brno Brno Czech Republic; ^3^ Department of Agroecology and Crop Production, Faculty of Agrobiology, Food and Natural Resources Czech University of Life Sciences Prague Praha – Suchdol Czech Republic; ^4^ Institute of Landscape Ecology, Bratislava Slovak Academy of Sciences Nitra Slovak Republic; ^5^ Crop Research Institute Prague Czech Republic

**Keywords:** elevation, host range, mid‐domain effect, parasitism rate, parasitoid distribution, parasitoid–host interaction

## Abstract

The evolutionary processes that shape host‐parasitoid coexistence in a changing environment are poorly understood. We examined the large‐scale distribution of highly specialised polysphinctine Darwin wasps associated with spiders along an elevational gradient and tested the hypothesis that distribution and parasitism rates depend on elevation, habitat type and the species and age composition of the host community. Further, on the basis of a large‐scale dataset, we examined the hypothesis that three‐dimensional webs in spiders may be an evolutionary adaptation against polysphinctine parasitoids. We found significant variation in parasitoid distribution and parasitism rates along a 1500 m elevational gradient in central Europe. The optimal model showed a humped shape for the parasitism rate on an elevational gradient. Overall, we found relatively low parasitism rates (4%) on spiders, with the highest parasitism rates in non‐forested riparian vegetation and the lowest in agroecosystems. Rates of parasitism varied significantly among spiders forming different types of webs (foraging guilds). Spiders spinning 3D webs were dominant in the spider community, but parasitism on them was lower compared to spiders spinning 2D webs, probably because of the defensive function of the 3D web architecture. The bottom‐up approach, in which the entire spider host community is analysed for parasitism rate, supports the hypothesis that 3D webs are evolutionarily novel and could have arisen as a result of the need for defence against enemies such as parasitoids.

## Graphical abstract

We examined the large‐scale distribution of highly specialised parasitoids associated with spiders along an elevational gradient and tested the hypothesis that distribution and parasitism rates depend on elevation, habitat type and the species and age composition of the host community. Further, on the basis of a large‐scale dataset, we examined the hypothesis that three‐dimensional webs in spiders may be an evolutionary adaptation against polysphinctine parasitoids.

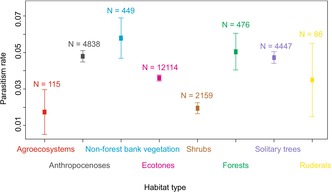

## 1 Introduction

The distribution of species is not random but determined by a wide range of abiotic and biotic environmental variables. Understanding the spatial distribution of a species is a central theme in biogeography (Li et al. 2009) and animal ecology (Wisz et al. 2013; Speed et al. 2019). The distributions of organisms on spatial scales can be traced along gradients of different environmental variables, including elevation (Miller 2010). Increasing elevation is often connected with a decrease in species richness (Terborgh and Weske 1975; Hunter and Yonzon 1993; McCain and Grytnes 2010) and at low‐ and mid‐elevations the species richness may differ among taxa and among regions (Rahbek 1995; McCain 2007). Rahbek (1995) summarised knowledge on elevational diversity patterns and found that most studies detected the highest species richness at lower elevations, as predicted by Rapoport's effect (Stevens 1992) and by the ‘Mid‐domain effect’ (Colwell and Lees 2000). Unimodal (hump‐shaped) distributions along an elevation gradient have already been observed in various organisms (e.g., Lenoir et al. 2008; Rahbek 1995; Sánchez‐Cordero 2001).

The development of living organisms is closely linked to the variables of the environment in which they live, especially organisms that develop directly in association with other organisms living in the same environment. One of the tightest forms of coexistence between organisms is found among foraging specialists, including predators and parasitoids and their prey or hosts. Such coexistence is conditioned by a balance in adaptations between the two sides. The balance of these adaptations on both sides of the interaction is crucial, as predator/parasitoid survival is dependent on adaptations to capture prey (Petráková et al. 2015) or to successfully oviposit on a host (Takasuka and Matsumoto 2011; Takasuka et al. 2018). On the other side of the interaction, the prey/hosts have evolved adaptations in response to the predation/parasitoid threat (Holen and Johnstone 2004; RobledoOspina and Rao 2022). The diversity of parasitoids associated with insect hosts significantly decreased with increasing elevation (Péré et al. 2013; Flinte et al. 2023); however, there is no detailed study on the effect of elevation on the distribution of parasitoids associated with spiders, which are an abundant and rich taxonomical group along the whole elevational gradient.

Elevation is among the most useful natural variables for testing the ecological and evolutionary responses of biota (Körner 2007) and is ideal for studying large‐scale distributions of species (Lomolino 2001). Such large‐scale data, encompassing both parasitoid and host distribution, may also reveal new patterns with respect to parasitoid–host coexistence and coevolution. Interacting taxa, e.g., those in host‐parasitoid systems, may not respond to elevation in the same manner (Maunsell et al. 2015). The distribution of parasitoids as a function of elevation is even more complex because it depends directly on host ecology and multidirectional interactions with the host (Sivinski et al. 2000). Here, we focus on interactions between highly specialised parasitoid Darwin wasps from the *Polysphincta* genus group (Ichneumonidea, Ephialtini), hereafter referred to as polysphinctines, and very specific hosts, namely spiders, which also pose a potential threat to ovipositing wasps.

Polysphinctines are koinobiont parasitoids that exclusively attack only spiders, dangerous predators of insects, including wasps (Eberhard and Gonzaga 2019). Polysphinctine females attack mostly juvenile spiders, but some taxa also attack adult spiders (Fitton et al. 1987). After the adult female lays an egg on the abdomen of the spider host, the larva feeds on the spider. Parasitised spiders continue to hunt, feed and repair their webs without any restrictions. This type of parasitisation does not kill the host immediately. While the larva is small, it remains attached to the spider abdomen, developing into the last larval stage. During this time, the spider host catches prey and can also moult with the parasitoid. Before pupating, the larva kills and consumes its host, then pupates. Adult parasitoids are free living and mobile, being capable of searching for hosts (Hoedjes et al. 2011). The host selection process is divided into 4 steps: host habitat location, host location, host acceptance and host suitability (Vinson 1976; Vet 1983). Some authors emphasise that selecting a habitat has evolutionary aspects. Parasitoids are firstly attracted not to a particular host but to a certain type of habitat. By selecting the habitat in which to search for the host and neglecting other areas, the parasitoid restricts the number of potential host species that it will find (Salt 1935). General habitat preference may be influenced by temperature, humidity, light intensity, wind and food sources, as well as the flying and crawling habits of the parasitoid (Read et al. 1970). Adult parasitoids prefer specific plant species on which they feed on flower nectar or honeydew (Townes 1958).

**FIGURE 1:**
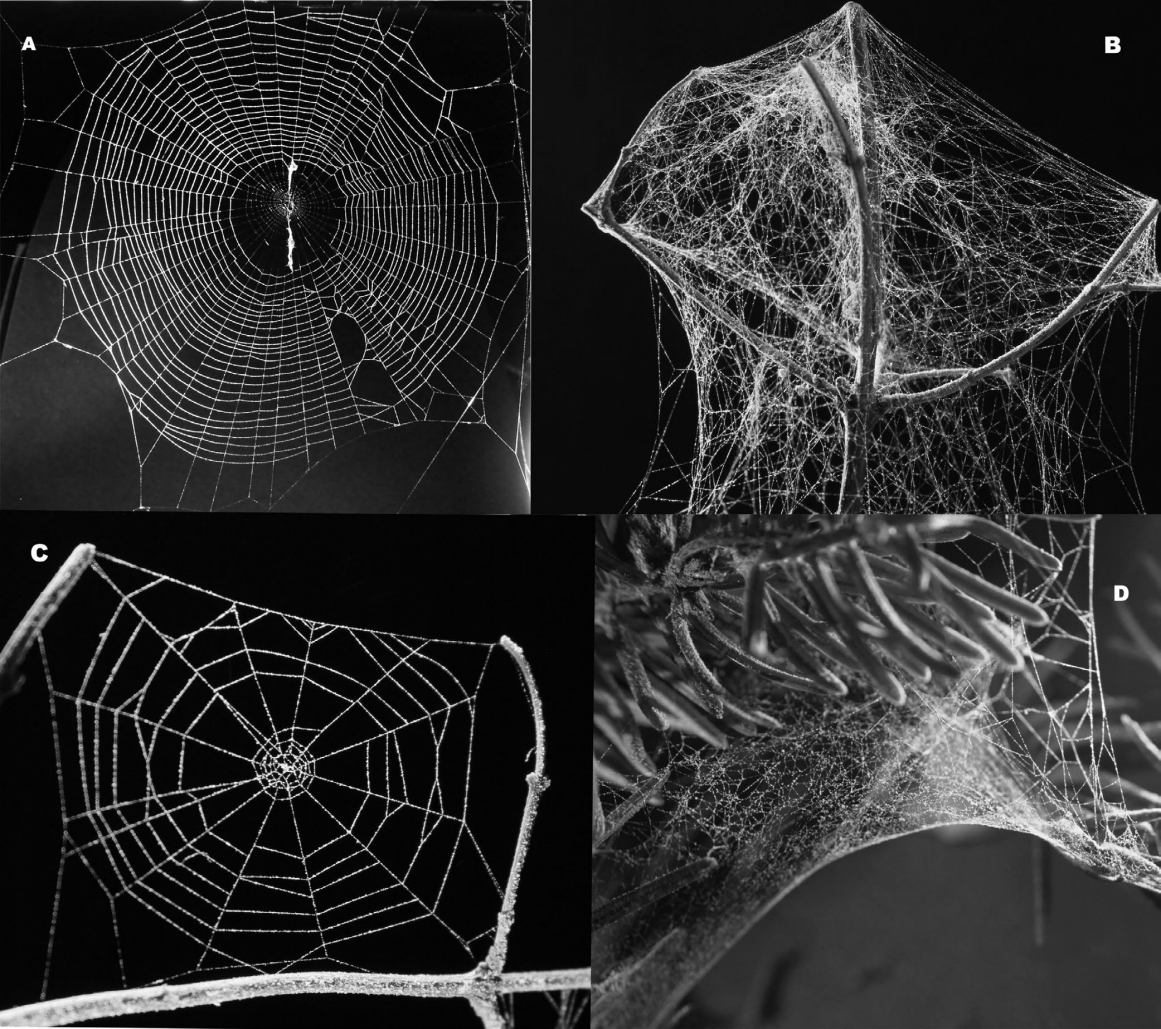
Types of spider webs analysed in the study. 2D web of araneid orb‐web weaver *Cyclosa conica* (A), 3D web of theridiid space web weaver *Theridion* sp. (B), 2D web of araneid orb‐web weaver *Araneus sturmi* (C), 3D web of linyphiid sheet web weaver *Mughiphantes mughi* (D).

Spiders differ in the hunting strategies they use. Some spiders are active hunters and others use webs of various types (e.g., 2‐dimensional vs. 3‐dimensional webs) (Blackledge et al. 2009). They are classified into guilds according to their foraging strategy (web type or hunting method), prey range, vertical stratification in their habitat, and circadian activity (Cardoso et al. 2011). In respect of functional diversity, aerial web‐building spiders can be distinguished into: 2D web weavers (who build orb webs; e.g., spiders of the family Araneidae and Tetragnathidae, Figure 1a,c), and 3D web weavers including space web weavers, e.g. Dictynidae and Theridiidae (who build silk tangles, Figure 1b) and sheet web weavers, e.g. Linyphiidae (who build horizontal capturing sheet webs surrounded by a silk tangle, Figure 1d) (e.g., Cardoso et al. 2011). Except for a few species from four evolutionarily ancestral genera (the *Schizopyga* subgroup), polysphinctines attack aerial web‐building spiders and their host specificity is very high (Matsumoto 2016). Each species of polysphintine wasp is only associated with spiders from one foraging guild and almost always from one family (Eberhard and Gonzaga 2019). Many of them are associated with only one genus (Korenko et al. 2014, 2015) or even only one spider species (Korenko et al. 2017). An important factor influencing the distribution of parasitoids and the rate of parasitism is the proportion of spiders of specific guilds in the local spider community. Along an elevational gradient, the proportion of specific foraging guilds in a spider community can vary considerably (Korenko et al. 2022).

Parasitoids generally exploit easily accessible prey with limited defences (various insects) (e.g., Godfray 1994). In contrast, spiders are dangerous hosts that use silk, web architecture and venom (with the primary use for catching prey) as defences against enemies, including parasitoids (e.g., Blackledge et al. 2003). Spiders are capable predators (Michalko et al. 2019) and, as hosts, can be dangerous to hymenopteran parasitoids (Kloss et al. 2016). Gonzaga et al. (2017) suggested that the wasp attack strategy depends on the specific host response and the architecture of host webs. To avoid spider attack during oviposition, polysphinctines have evolved unique behaviours that are fine‐tuned to the web design of a particular host spider (e.g., Eberhard 2000; Kloss et al. 2016; Takasuka and Matsumoto 2011; Takasuka et al. 2009, 2018). Wasps can use aggressive mimicry by mimicking struggling prey to lure the spider out of its hiding place (Takasuka and Matsumoto 2011; Takasuka et al. 2018; Eberhard 2000). These behavioural adaptations allow polysphinctines to specialise on 3D web‐building spiders that are hidden inside their webs and shelters. On the other hand, such high specialisation probably eliminates host transfer outside of a preferred foraging guild and a specific type of web.

A three‐dimensional spider web is expected to be an effective defence against generalist parasitoids—specifically, predatory wasps (Blackledge et al. 2003). The general validity of this hypothesis was questioned by Uma and Weiss (2010), whose behavioural experiments clearly demonstrated that chemical cues, rather than web architecture, mediate prey recognition by predatory wasps. However, the results from these two studies do not contradict each other but show that understanding the evolution of spider webs requires a holistic approach. Both studies analysed sphecid predatory wasps, which are idiobionts and generalists that attack a wide spectrum of spiders including 2D and 3D web builders. It is questionable whether the protection offered by 3D spider web structures could also be effective against highly specialised parasitoids such as polysphinctines. High species‐level specialisation in the process of coevolution with hosts has also allowed polysphinctines to invade 3D web weavers in their well‐protected webs. A large‐scale assessment of spider communities and a comparison of the dominance of particular guilds and the parasitism rates found among them could show us whether polysphinctines attack 2D web builders more often than 3D web‐building spiders. The importance of elevation in spider‐parasitoid coevolution could be expected in cases where the compositions of spider communities (e.g., the proportions of 2D vs. 3D web weavers in the communities) differ considerably along the elevation gradient.

Our main objective was to investigate the distribution, host range and parasitism rate of highly host‐specific polysphinctine parasitoids along an elevational gradient. We tested the following hypotheses: (1) both the distribution and parasitism rate differ along the elevational gradient, (2) the parasitism rate differs among the foraging guilds of spiders, (3) among habitats and (4) among developmental stages. Further, we used the large‐scale data set obtained to understand the function of web architecture in parasitoid–spider host coevolution. We tested the hypothesis formulated by Blackledge et al. (2003), which hypothesised selection pressure from generalist parasitoids on the evolution of spider web architecture. Our study asked whether this selection pressure can also be assumed from highly specialised parasitoids.

## 2 Material and Methods

### 2.1 Collection of Data

**FIGURE 2:**
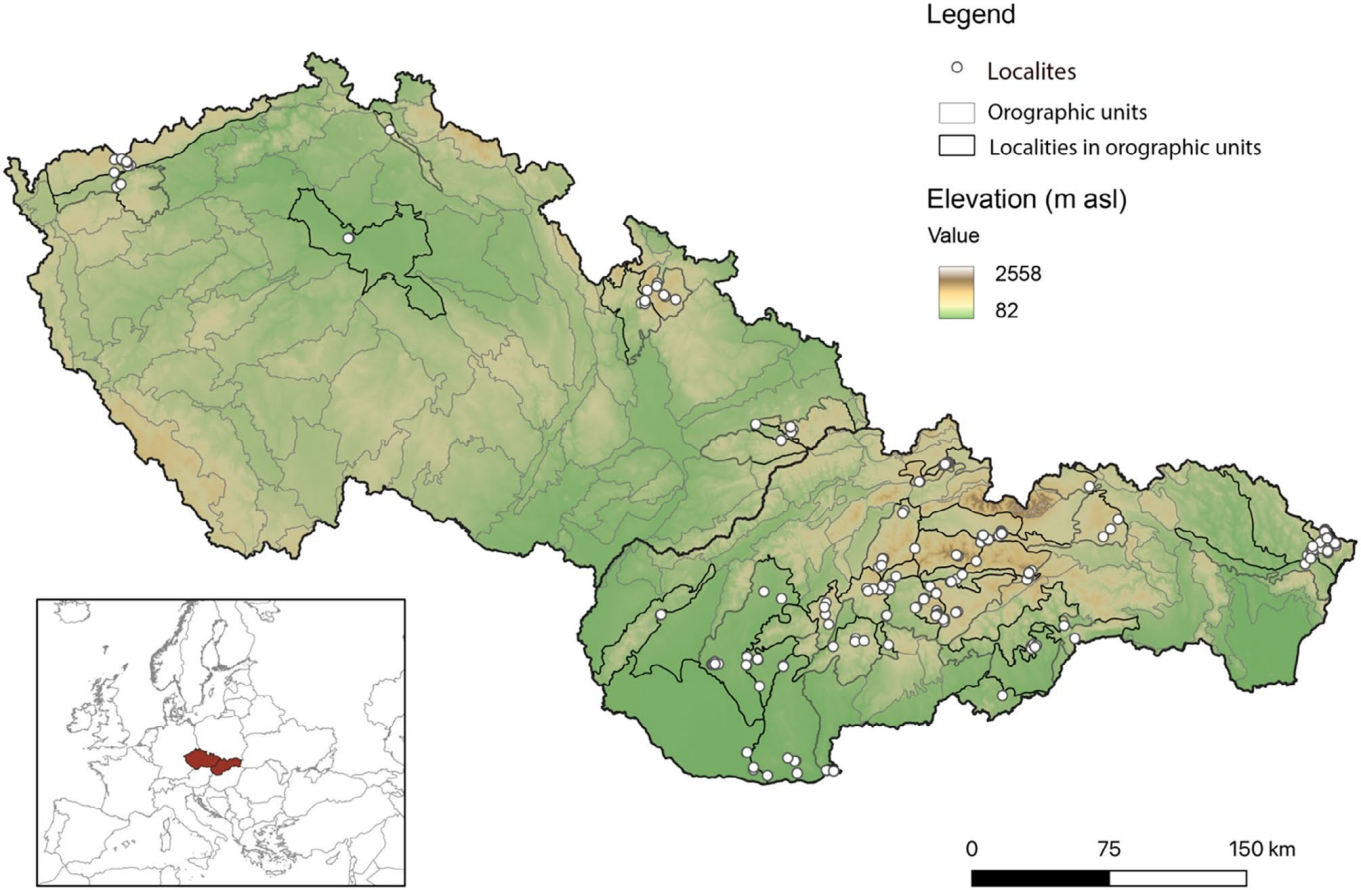
Map of 175 localities in 36 orographic units in central Europe (specifically, in the Czech Republic and Slovakia).

Assemblages of arboreal spiders (potential hosts of wasps from the *Polysphincta* genus group) were studied in 175 localities in 36 orographic units in Central Europe (the Bohemian Massif and Western Carpathians on the territory of the Czech Republic and Slovakia) along an elevational gradient from 111 to 1654 m a.s.l. in the years 2016–2023 (Appendices S1 and S2; Figure 2). In the studied area, we collected specimens in localities at different elevational levels from the lowest to the highest elevation. Three samplings at one time were performed at each locality. Spiders were collected by beating the branches of tree canopies (between 30 and 250 cm above the ground), with a square‐shaped beating net (1‐m^2^ area) placed beneath. One sampling was conducted along a 50‐m transect of trees or shrubs, depending on the type of habitat. About 1 h was allocated for the collection of each sample. Parasitoid larvae were visually identified on the sampled spiders (attached on the spider opisthosoma). The parasitised spiders were maintained individually in tubes under controlled environmental conditions in the laboratory. They were fed with fruit flies ( *Drosophila melanogaster* ) and kept for wasp rearing and future identification. Parasitoid larvae that were not detected in the field were identified on spiders from 95% ethanol samples prepared in the laboratory for analyses of the compositions of spider communities. Specimens of spider hosts were identified to species level whenever possible following Nentwig et al. (2024) using the nomenclature in the WSC (2024). For the identification of wasps, we used the determination key by Fitton et al. (1988) and Zwakhals (2006). In the analysis of the effect of habitat on parasitism rates, we used the categorisation of habitats according to the Catalogue of Slovak Biotopes (Stanová and Valachovič 2002), which is representative for central Europe. To determine the effect of spider foraging guild on parasitism rates, we used the classification of foraging guilds by Cardoso et al. (2011): respectively, the guilds Orb‐web weavers, Space web weavers and Sheet web weavers.

### 2.2 Statistics

We used only spiders from potential host species in the analyses, i.e. web‐building spiders from the preferred spider host family. In Europe, polysphinctine wasps are strictly associated only with one spider family (e.g., Fitton et al. 1987). We used generalised additive mixed effect models (GAMMs) from the R package ‘mgcv’, because the relationship with elevation may be non‐linear (e.g., Roslin et al. 2017). The non‐linear effect of elevation was modelled by thin‐plate regression spline with an implicitly set number of knots, i.e. 10. We used GAMM with a binomial error structure (GAMM‐b), as the data had Bernoulli's distribution (Pekár and Brabec 2019). There was no problem with overdispersion (Scale parameter = 1.00). The orographic units acted as random effects. We used an information theoretic approach to select the optimal model (Zuur et al. 2015). This approach selects the most influential variables objectively because all models are compared simultaneously. We built 16 competing models, including a null model (Table 2), where we combined the additive effects of spider web type (Orb web, Sheet web and Space web), habitat type (Agroecosystems, Anthropocenoses, Non‐forest bank vegetation, Ecotones, Scrubs, Forests, Soloist trees and Ruderals) and elevation. We selected the most parsimonious model with the lowest Akaike Weights (AIC). The Mann–Whitney Test was used to find differences in the abundances of 2D vs. 3D building spiders at specific elevations.

## 3 Results

### 3.1 Potential Host Range

A total of 235 spider species from 26 families (in total, 44,216 specimens) were collected by beating tree and shrub branches in 175 localities in Central Europe. The most abundant spiders were from the family Philodromidae (*N* = 8793), followed by the family Theridiidae (*N* = 8762) and Araneidae (*N* = 7204). The community of potential hosts (taxa from these families already known to be attacked by wasps of the *Polysphincta* genus group) included 24,801 individuals belonging to the families Araneidae (orb‐web weavers), Dictynidae (space web weavers), Linypiidae (sheet web weavers), Tetragnathidae (orb‐web weavers) and Theridiidae (space web weavers) (Appendix S2).

### 3.2 Realised Host Range

We collected 951 parasitised spiders, and 13 species of polysphinctine wasps were reared from them (Table 2). We found that all parasitoid taxa were associated only with spiders from one specific family and from one specific foraging guild. Parasitised spiders (*N* = 951) represented only 4% of potential hosts, but the parasitism rate was significantly larger than zero (GAMM‐b, *p* < 0.001). The parasitised spiders belonged to five families, which all belonged to the group of expected hosts. This group represented only 15% of all collected spider families. The degree of parasitism on spider families was in the following order, starting with the highest: Araneidae (*N* = 452), Theridiidae (*N* = 261), Tetragnathidae (*N* = 103), Dictynidae (*N* = 72) and Linyphiidae (*N* = 63). Orb‐web‐building spiders from the family Araneidae were hosts of wasps from the genera *Reclinervellus*, *Sinarachna* and *Polysphincta* and one species of *Zatypota* (*Z. picticollis*). The family Araneidae was represented by a total of 14 genera and 29 species, but only four genera with 9 araneid species were accepted as hosts (Table 1). Orb‐web‐building spiders from the family Tetragnathidae were hosts of two wasps from the genera *Acrodactyla* (the *quadrisculpta* group)—respectively, *A. carinator* and *A. rufithorax* . The family Tetragnathidae was represented by a total of 3 genera and 9 species. Only the genus *Tetragnatha* was accepted as a host (Table 1). Space web‐building spiders from the family Theridiidae and Dictynidae were exclusively hosts of wasps of the genus *Zatypota*. *Z. albicoxa* and *Z. percontatoria* were associated with Theridiidae and *Z. anomala* with Dictynidae. The family Theridiidae was represented by a total of 23 genera and 36 species. Only six genera with nine theridiid species were accepted as hosts (Table 1). The family Dictynidae was represented by five genera, of which only *Dictyna* and *Nigma* with four species were observed as hosts of *Z. anomala* (Table 1).

**TABLE 1:** The parasitoid community and associations with hosts.

Parasitoid	Hosts	Family	Guild^a^	*N*
*Acrodactyla degener* (Haliday, 1838)	*Entelecara* sp., *Mughiphantes mughi* (Fickert, 1875), *Neriene* sp., *N. peltate* (Wider, 1834), *Neriene radiata* (Walckenaer, 1841), *Kaestneria dorsalis* (Wider, 1834)	Linyphiidae	Sheet web	63
*Acrodactyla carinator* (Aubert, 1965)	*Tetragnatha* sp., *Tetragnatha montana* Simon, 1874, *T. pinicola* L. Koch, 1870	Tetragnathidae	Orb web	101
*Acrodactyla rufithorax* (Györfi, 1944)	*Tetragnatha* sp.	Tetragnathidae	Orb web	2
*Polysphincta boops* Tschek, 1868	*Araniella* sp., *Araniella alpica* (L. Koch, 1869), *Araniella cucurbitina* (Clerck, 1757)	Araneidae	Orb web	14
*Polysphincta tuberosa* (Gravenhorst, 1829)	*Araneus diadematus* Clerck, 1757, *Araneus marmoreus* Clerck, 1757, *Araneus sturmi* (Hahn, 1831), *Araniella alpica* (L. Koch, 1869), *Araniella cucurbitina* (Clerck, 1757), *Araniella opisthographa* (Kulczyński, 1905), *Cyclosa conica* (Pallas, 1772)	Araneidae	Orb web	179
*Polysphincta tuberosa* group^b^	*Araniella* sp., *Araniella alpica* (L. Koch, 1869), *Araniella cucurbitina* (Clerck, 1757), *Araniella opisthographa* (Kulczyński, 1905)	Araneidae	Orb web	65
*Reclinervellus nielseni* (Roman, 1923)	*Cyclosa conica* (Pallas, 1772)	Araneidae	Orb web	1
*Sinarachna nigricornis* (Holmgren, 1860)	*Araneus sturmi* (Hahn, 1831), *Araneus triguttatus* (Fabricius, 1775)	Araneidae	Orb web	136
*Sinarachna pallipes* (Holmgren, 1860)	*Araniella* sp., *Araniella cucurbitina* (Clerck, 1757), *Araniella opisthographa* (Kulczyński, 1905)	Araneidae	Orb web	42
*Zatypota albicoxa* (Walker, 1874)	*Parasteatoda* sp.	Theridiidae	Space web	1
*Zatypota anomala* (Holmgren, 1860)	*Dictyna* sp., *Dictyna arundinacea* (Linnaeus, 1758), *Dictyna uncinata* Thorell, 1856, *Nigma flavescens* (Walckenaer, 1830), *Nigma walckenaeri* (Roewer, 1951)	Dictynidae	Space web	72
*Zatypota discolor* (Holmgren, 1860)	*Phylloneta* sp.	Theridiidae	Space web	2
*Zatypota percontatoria* (Muller, 1776)	*Neottiura bimaculata* (Linnaeus, 1767), *Paidiscura pallens* (Blackwall, 1834), *Phylloneta impressa* (L. Koch, 1881), *Phylloneta sisyphia* (Clerck, 1757), *Platnickina tincta* (Walckenaer, 1802), *Theridion* sp., *Theridion boesenbergi* Strand, 1904, *Theridion pinastri* L. Koch, 1872, *Theridion varians* Hahn, 1833	Theridiidae	Space web	258
*Zatypota picticollis* (Thomson, 1888)	*Araneus diadematus* Clerck, 1757, *Cyclosa conica* (Pallas, 1772), *Gibbaranea* sp., *Gibbaranea omoeda* (Thorell, 1870)	Araneidae	Orb web	15

^a^ Guild means a foraging guild of a spider host as determined by a specific web type.

^b^ *Polysphincta tuberosa* group includes wasps of the genus *Polysphincta*, including mostly *P. tuberosa* , possibly *P. boops* .

Sheet web‐building spiders from the family Linyphiidae were hosts of the wasp *Acrodactyla degener* (the *A. degener* group). In linyphiids, there were two major subfamilies: Micronetinae and Erigoninae. Species in Micronetinae belong to the web‐building guild, so we examined this subfamily only. Potential spider hosts included a total of 30 genera and 46 species; however, only four genera with five species were observed as hosts (Table 1).

### 3.3 Parasitoid Distribution and Trait Preference

The most optimal model included the additive effects of foraging (web type), development stage, habitat type and elevation (Table 2).

**TABLE 2:** Comparison of models investigating the factors determining the parasitism rate. The intrinsic factors related to spiders were the web type (web; Orb web, Sheet web and Space web) and stage and sex (juvenile/adult, female/male). The extrinsic factors were habitat type (Agroecosystems, Anthropocenoses, Non‐forest bank vegetation, Ecotones, Shrubs, Forests, Solitary trees, Ruderals) and elevation (masl). The most optimal model is highlighted in bold.

Model	AIC
**web + stage + habitat + masl**	**7915.2**
web + habitat + masl	7935.2
web + stage + masl	8008.5
web + habitat + stage	7924.1
stage + habitat + masl	7983.8
web + stage	7942.3
web + habitat	8018.4
web + masl	8037.1
stage + habitat	7994.1
stage + masl	8003.1
habitat + masl	8113.2
web	8044.3
stage	8012.2
habitat	8124.5
masl	8142.0
null	8150.9

**FIGURE 3:**
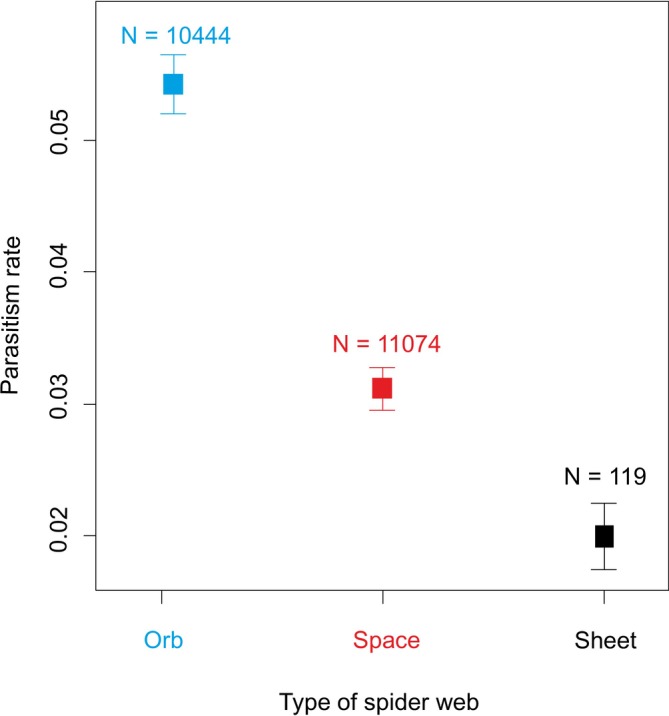
Comparison of parasitism rates by parasitoid wasps among spider web types. The points show raw means across the whole studied elevation gradient; the lines are SE.

The parasitism rate differed significantly among host foraging guilds (web types) (GAMM‐b, $\chi_{2}^{2}$ = 69.7, *p* < 0.001, Figure 3). Respectively, orb‐web spiders exhibited the highest parasitism rate (contrasts between web types, *z*‐value > 5.9, *p* < 0.001), followed by space‐web spiders (contrasts between web types, *z*‐value > 2.4, *p* < 0.015), while sheet‐web spiders exhibited the lowest parasitism rate. Spiders with 2D webs (orb‐webs) were parasitised at a higher frequency compared to spiders with 3D webs (space‐ and sheet‐webs): respectively, 6.19% vs. 3.54%.

**FIGURE 4:**
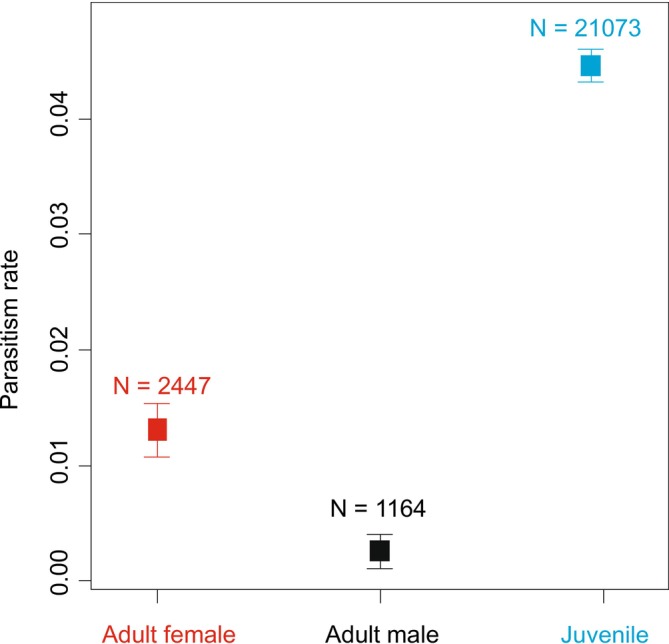
Comparison of parasitism rates by parasitoid wasps among ontogenetic stages and sexes of web‐building spiders. The points are raw means across the whole elevation gradient and the lines show SE.

The development stage also significantly influenced the parasitism rate (GAMM‐b, $\chi_{2}^{2}$ = 52.1, *p* < 0.001; Figure 4). Juveniles were the most parasitised stage (contrasts between stages, *z*‐value > 4.6, *p* < 0.001) and females were parasitised more than males (contrasts between stages, *z*‐value = 2.8, *p* = 0.006).

**FIGURE 5:**
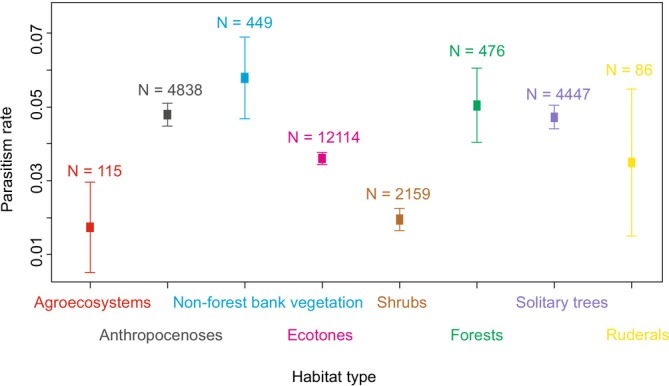
Comparison of parasitism rates by parasitoid wasps among eight habitat types. The points show raw means across the whole elevation gradient and the lines are SE.

The habitat type significantly influenced the parasitism rate (GAMM‐b, $\chi_{7}^{2}$ = 30.2, *p* < 0.001; Figure 5); it was the highest for non‐forest bank vegetation and the lowest for agroecosystems.

**FIGURE 6:**
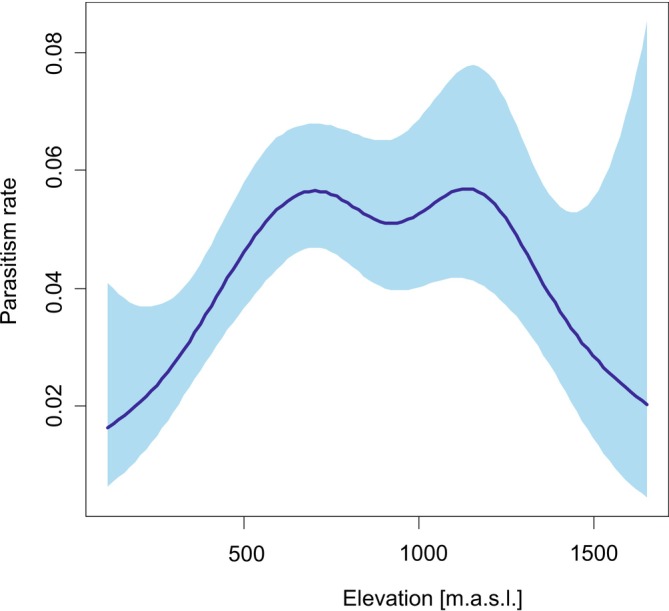
The hump‐shaped relationship between parasitism rate and altitude. The thick line is the estimated relationship, and the band is 95% CI. The estimated relationship and its 95% CI are for juvenile orb‐weaving spiders in ecotones, as these spiders were parasitised the most (Figures 2 and 3) and because ecotones had the largest sample size (Figure 4). The non‐linear effect of elevation was modelled by thin‐plate regression spline.

**FIGURE 7:**
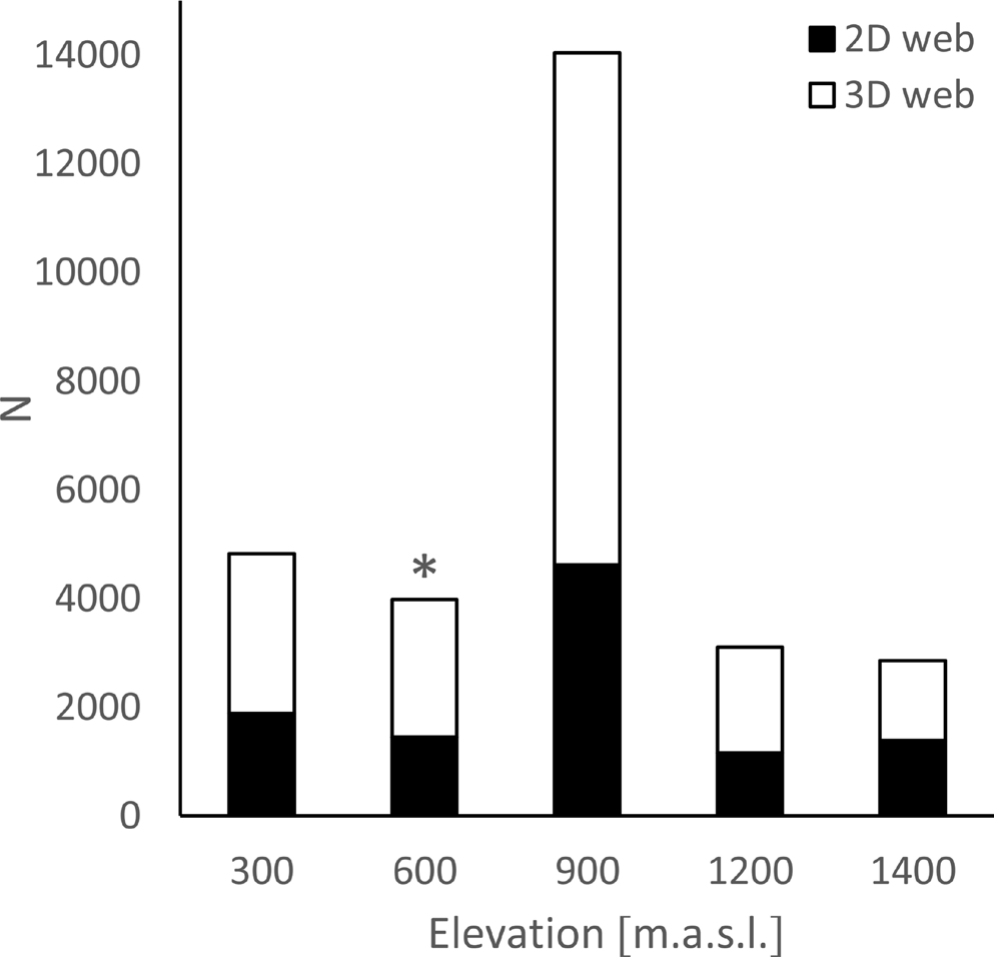
The total proportions of 2D and 3D spiders in the community of potential hosts (web‐building spiders) at specific altitude ranges. The number in the box is the number of individuals. Asterix means a statistical significance of *U* = 1188.5, *p* = 0.014.

There was a significant hump‐shaped relationship between parasitism rate and elevation (GAMM‐b, edf = 4.6, *χ* ^2^ = 15.2, *p* = 0.034; Figure 6). The total proportion of 2D and 3D spiders in the community of potential hosts differed across all elevations (Figure 7), but statistically significant differences were only in the elevation category up to 600 m a.s.l. (300–600 m a.s.l.) (U = 1188.5, *p* = 0.014).

## 4 Discussion

This study provides valuable insights into the distribution and ecological interactions of polysphinctine parasitoids and their spider hosts along an elevational gradient in central Europe. The findings highlight the significance of environmental factors in shaping parasitism rates within spider communities and contribute to a broader understanding of the evolution of this parasitoid–host system.

### 4.1 Elevation and Habitat Traits

We found a hump‐shaped relationship in the distribution of polysphinctine parasitoids (expressed by the rate of parasitism) across an elevational gradient. This could be related to the preference of spiders for mid‐elevations (e.g., Bosmans et al. 1986; Chatzaki et al. 2005). Results from a study of gnaphosid spiders showed that species richness declines with elevation and follows a hump‐shaped pattern (Chatzaki et al. 2005). Other studies of spider diversity along an elevational gradient showed monotonic declines (Yanoviak et al. 2003; González‐Reyes et al. 2017). Multiple taxa studies analysed plant and animal communities (Peters et al. 2016) and demonstrated that in 44% of all tested taxa (including spiders and parasitoid wasps) diversity patterns were of a unimodal nature. The distribution of parasitoids is not random but is influenced by spatial and other processes that lead to the aggregation of parasitoids (Hassell 2000). We found that elevation is a significant variable in the distribution of parasitoids, because it follows the distribution of their host, as was also observed in other studies (Virtanen and Neuvonen 1999; Aguirre et al. 2018; Korenko et al. 2022). The distribution on an elevational gradient varies among both parasitoid taxa and preferred host taxa. The number of Doryctinae parasitoids declined with elevation in response to their host distribution, where wood‐boring beetles were distributed mostly at low elevation. In contrast, parasitoids in the family Braconinae showed two elevational peaks due to changes in host use, ranging from wood‐boring beetles at low elevation to butterflies and flies at higher elevation (Aguirre et al. 2018). The higher richness of parasitoids at low elevations (< 500 m) was documented in Costa Rica despite their hosts preferring higher elevations (up to 1600 m). In this case, the effect of temperature may have been stronger than that of elevation or host distribution (e.g., van der Ent and Shaw 1998).

Other significant variables appear to be habitat traits, such as temperature, humidity, light intensity, wind, food sources and others, which vary along an elevational gradient. Humidity is the limiting factor for Darwin wasps (Townes 1958). We found that parasitism decreased at low elevations, which represent warmer and drier habitats and that parasitism was the highest in non‐forest bank vegetation characterised by high humidity. Obviously, there are dominant parasitoid species that are associated with spider species preferring more humid environments, such as wasps of the genera *Acrodactyla* associated with spiders of the genus *Tetragnatha*, of which most species occur in humid habitats.

Another important factor in species dispersal is human activity (Cardoso et al. 2020). Anthropogenically influenced landscapes are known to promote lower diversity (e.g., Branco and Cardoso 2020; Plath et al. 2021). We found the lowest parasitism rates in agroecosystems, which is probably a consequence of the negative effects of human agricultural activities.

### 4.2 Host Stage Preference

As shown above, the most frequently parasitised developmental stage is the juvenile stage in central Europe. This preference is also known from the literature (e.g., Korenko et al. 2014; Fritzén and Shaw 2014). Among adults, females were parasitised more frequently than males. The short lifespans of adult male spiders exclude them as suitable hosts for the development of parasitoid larvae (Fitton et al. 1987). Although in the Temperate zone with changing seasons, the preference for juvenile spiders is the rule for polysphinctines (e.g., Korenko et al. 2011), in the Neotropics several polysphinctines prefer subadult and adult female spiders (Barrantes et al. 2008; Eberhard and Gonzaga 2019). This is certainly linked to the significantly larger wasp sizes in the Neotropics and consequently a preference for larger—i.e., subadult or adult—spiders. Also, there is no winter period in the Neotropics. In Europe, polysphinctines overwinter mostly as larvae on juvenile spiders (Fitton et al. 1987) and cannot survive the winter on adults (Fitton et al. 1987) because adult male and female spiders do not survive the winter in most species (Nentwig et al. 2024).

### 4.3 Parasitism Rates

Parasitised spiders have been shown to represent only 4% of potential hosts, which is not a high number. We found large differences in parasitism within a locality, where parasitism was high on some trees while no spiders were parasitised on neighbouring trees. Eberhard (2019) expressed this in terms of clumped searching behaviour by the wasps. We assume that the female needs to hatch eggs and therefore lays a higher number of eggs in the first locality where hosts are available; e.g., on one tree, we collected several parasitised spiders, while on other trees we did not find a single parasitised individual. The parasitism rate can differ considerably among spider taxa. For example, Barrantes et al. (2008) found substantial differences in the parasitism rate between the spiders *Theridion evexum* Keyserling, 1884, with a parasitism rate of 1.39% ± 1.80% and *Allocyclosa bifurca* (McCook, 1887), with a parasitism rate of 7.8% ± 7.6%. This agrees with our results, and this variability in the parasitism rate among species may explain the variability in the parasitism rate among localities. In central Europe, the parasitism of polysphinctines is low, as shown by Korenko et al. (2011), where in a 2‐year study the parasitism rate of *Z. percontatoria* was only 1.74% (*N* = 4814) and 0.83% (*N* = 2539), respectively. Our results also document a low parasitism rate, but locally, parasitism rates can be high, as observed in A. degener with local parasitism rate above 10% in montane areas in Central Europe (Sýkora, pers. obs.).

### 4.4 Parasitoid Selection Pressure on Web Architecture Evolution

Our robust dataset on the distributions of spiders and their parasitoids revealed that parasitism differs significantly among spiders forming different types of webs—respectively, different foraging guilds. 2D‐web‐building spiders were significantly more parasitised than 3D‐web‐building spiders, although potential spider hosts building 3D webs were more abundant in the spider community compared to spiders building 2D webs. A similar preference for 2D‐web weavers was found in sphecid wasps by Blackledge et al. (2003). Sphecids are idiobiont parasitoids, whereas the polysphinctines analysed here are koinobionts, and, therefore, their evolutionary histories could differ considerably. Idiobiont parasitoids fully paralyse their hosts during oviposition, whereas koinobionts allow their hosts to continue to develop after oviposition (Askew and Shaw 1986). The oviposition strategy determines host utilisation and is inherently related to the characteristics of host–parasite coevolution. Sphecids attack a wide range of spiders and can be considered generalists compared to polysphinctines, whose host range is very specific.

Blackledge et al. (2003) hypothesised that generalist idiobiont sphecid wasps, as one of the main predators of orb‐web spiders, exert directional selection pressure on the construction of three‐dimensional webs. The question is, what selection pressure is exerted by specialist parasitoids? The host spectrum of specialists is strictly given (e.g., Godfray 1994) and the acceptance of new hosts in polysphinctines is very rare and only occurs under specific conditions, or it happens accidentally (Korenko, pers. observ.). A top‐down study (in which higher trophic level organisms—parasitoids—are studied, and only currently accepted hosts are included in the study of their interactions) alone cannot currently reveal the history of host specificity nor identify the host range of ancestral species. Therefore, we performed our analysis in the bottom‐up direction, in which the entire spider community was analysed for parasitism rate. Hence, host preference at the entire taxonomic group level (i.e., for polysphinctines) was estimated. The entire spider community represents all potential hosts that have evolved up to the present and are available for polysphincta wasps as hosts in shared habitats. This approach allowed us to consider the hypothesis of a selection pressure from highly specialised parasitoids on the evolution of spiders and the architecture of their webs. Complex evolutionary innovations, such as the three‐dimensional space or sheet web, have undoubtedly evolved for many reasons, and we suggest that selection pressure from parasitoids is one of them. Both sphecid generalists (Blackledge et al. 2003) and polysphinctine specialists (this study) significantly prefer 2D web‐building spiders as their hosts.

The defensive function of 3D webs against parasitoids/predators and their evolution is not clear. Indeed, as Uma and Weiss (2010), for example, suggest, the preference for 2D spiders is determined by chemical signals and the web itself is not important for host acceptance. There is an unambiguous chemical difference between the silk of 2D spiders and 3D spiders (e.g., Schulz 2004; Uma and Weiss 2010). Our results do not refute this hypothesis that wasps prefer 2D spider webs because they are attracted to the chemical signals they produce. This preference was programmed during their long coevolution with 2D web weavers, which lasted from the early Cretaceous, when sphecids first evolved and diversified (Bohart and Menke 1976). Also, the results of Uma and Weiss (2010) do not refute the importance of parasitoids in the evolution of spider webs, because it is quite possible that the preference for specific cues evolved only after the diversification of the web architecture had evolved.

Two‐dimensional webs first evolved and diversified in the Jurassic; 3D webs evolved and diversified much more recently in the Cretaceous and Cenozoic (Vollrath and Selden 2007; Penney and Selden 2002). The origin and diversification of ancestral spider parasitoids (both, generalist sphecid wasps and highly specialised polysphinctine wasps) occurred before the Cretaceous‐Paleogene boundary (e.g., Bohart and Menke 1976; Spasojevic et al. 2021); thus, parasitoids were able to coevolve with spiders and their webs, and the directional selection pressure on the construction of three‐dimensional webs, as a defensive innovation, could have arisen (Blackledge et al. 2003). Polysphinctines have had enough time to evolve high specificity and a koinobiont strategy; in contrast, sphecids remain as idiobiont parasitoids of spiders up to the present. The sphecid idiobiont life strategy is presumably linked with their generalist foraging strategy. Other common spider parasitoids are pompilid wasps, which are primarily idiobionts, but in a few taxa koinobiontism has evolved (e.g., Souza et al. 2015; Benamú et al. 2020). The origin of pompilids is much more recent and most of the extant subfamilies originated from the Paleogene (late Eocene to the Oligocene) (Waichert et al. 2015). The explanation may be that koinobiontism can also evolve in such a relatively short time, or that koinobiont lineages of pompilid wasps have a considerably longer history than expected. Although most pompilids are associated with ground‐dwelling spiders, they also attack web‐building spiders, so some impact on spider web evolution can also be expected. In short, selection pressure on the evolution of web architecture has been significantly influenced by spider parasitoids, but to understand this, a study across all taxonomic groups of spider‐associated parasitoids is required.

## 5 Conclusion

In this study, the first of its kind to study spiders as dangerous hosts in such detail, we revealed the distribution of spider‐associated polysphinctine parasitoids along an elevational gradient in central Europe and identified important variables that influence parasitism rates within the spider community. In addition, our findings also contribute to our understanding of the coevolution of polysphinctine parasitoids and their spider hosts and the related selection pressures on spider web evolution. In particular, the finding that interactions between specialised parasitoids and their hosts need to be studied by both top‐down and bottom‐up approaches in order to understand the evolution of the parasitoid–host system provides a new direction and methodological recommendation for the study of coevolution.

## Author Contributions

**Ľudmila Černecká:** conceptualization (equal), investigation (equal), methodology (equal), writing – original draft (lead), writing – review and editing (equal). **Radek Michalko:** formal analysis (equal), methodology (equal), software (equal), writing – original draft (equal). **Jakub Sýkora:** investigation (equal). **Peter Gajdoš:** data curation (equal), investigation (equal). **Pavol Purgat:** investigation (equal). **Kamil Holý:** investigation (equal). **Martina Dorková:** investigation (equal). **Stanislav Korenko:** conceptualization (equal), formal analysis (equal), funding acquisition (equal), investigation (equal), methodology (equal), project administration (equal), resources (equal), writing – original draft (equal), writing – review and editing (lead).

## Conflicts of Interest

The authors declare no conflicts of interest.

## Supporting information


**Appendix S1** Summary of studied localities included in analyses.

**Appendix S2** Number (*N*) and Parasitism rate (*P*) of collected spiders collected in the study.

## Data Availability

The authors confirm that the general data supporting the findings of this study are available within the article and Supporting Information. Detailed data and the database used are available on Dryad, a platform for publishing open data as Primary Data—parasitoid‐spider ECE‐2024‐11‐02349, link: https://doi.org/10.5061/dryad.9kd51c5tj. URL: http://datadryad.org/stash/share/v‐bNHc‐_fh1a3xRmvn0Pur5ba5bV_nu_L21VbFlWMI8.
