# Supplementary material for: A Holistic Approach to Parasitoid–Host Interaction Along an Elevational Gradient Revealed Coevolution Driven by Host Foraging Strategy
Source: Ecol Evol. 2025 Apr 11;15(4):e71227. doi: 10.1002/ece3.71227 (PMC11991926; doi:10.1002/ece3.71227)
Supplement: Supplementary file 1 — Appendix S1 Summary of studied localities included in analyses. [file ECE3-15-e71227-s001.docx]

| **Orographic unit (country)** | **N** | **Elevation**  **(m a.s.l.)** | **Sampling**  **(month/year)** |
| --- | --- | --- | --- |
| Beskydské predhorie (SR) | 3 | 249-288 | 9/2020, 5/2021 |
| Beskydy (CR) | 6 | 373-1035 | 5/2020 |
| Bukovské vrchy (SR) | 11 | 381-1002 | 9/2019, 5/2020, 9/2020, 5/2021 |
| Burda (SR) | 5 | 112-167 | 5/2020, 7/2020, 8/2020, 10/2020, 11/2020 |
| Cerová vrchovina (SR) | 1 | 329 | 8/2019 |
| Horehronské podolie (SR) | 3 | 534-823 | 10/2018, 11/2018, 10/2020 |
| Hronská pahorkatina (SR) | 7 | 110-205 | 7/2020, 8/2020, 10/2020, 11/2020, 2/2021, 3/2021, 4/2021, 5/2021 |
| Jeseníky (CR) | 12 | 480-1381 | 5/2020, 6/2020 |
| Jizerské Hory (CR) | 1 | 960 | 6/2020 |
| Kozie chrbty (SR) | 12 | 646-782 | 4/2018, 5/2018, 7/2018, 9/2019, 10/2018, 6/2020, 9/2020, 10/2020, 9/2021 |
| Kremnické vrchy (SR) | 3 | 831-1009 | 9/2019, 10/2019, 5/2020, 6/2020, 8/2020, 9/2020, 3/2021, 4/2021 |
| Krušné Hory (CR) | 12 | 486-1244 | 6/2020 |
| Laborecká vrchovina (SR) | 5 | 272-407 | 5/2020, 9/2020, 5/2021 |
| Levočské vrchy (SR) | 3 | 554-889 | 4/2021 |
| Liptovská kotlina (SR) | 3 | 687-802 | 9/2020, 10/2020, 9/2021 |
| Litavská pahorkatina (SR) | 2 | 138-248 | 11/2020, 3/2021, 4/2021 |
| Malé Karpaty (SR) | 1 | 255-288 | 9/2018 |
| Mělnická kotlina (CR) | 12 | 179-183 | 10/2020, 10/2021 |
| Nitrianska pahorkatina (SR) | 8 | 131-260 | 6/2020, 9/2020, 10/2020, 12/2020, 2/2021, 3/2021, 4/2021, 5/2021, 6/2021 |
| Nízke Tatry (SR) | 8 | 697-1654 | 8/2020, 9/2020, 10/2020, 9/2021 |
| Oravská kotlina (SR) | 3 | 714-812 | 5/2021, 6/2021 |
| Oravská Magura (SR) | 3 | 780-1300 | 6/2021, 9/2021, 10/2021 |
| Pliešovská kotlina (SR) | 1 | 464 | 6/2018, 6/2019, 6/2020 |
| Podunajská rovina (SR) | 1 | 112 | 5/2021, 7/2021 |
| Poľana (SR) | 5 | 1023-1368 | 10/2019, 5/2020, 6/2020, 7/2020, 4/2021, 5/2021 |
| Revúcka vrchovina (SR) | 1 | 298 | 5/2021, 6/2021 |
| Rimavská kotlina (SR) | 4 | 214-318 | 3/2021, 5/2021, 6/2021, 8/2021 |
| Slovenský kras (SR) | 2 | 495-663 | 6/2020 |
| Spišská Magura (SR) | 1 | 698 | 6/2018 |
| Starohorské vrchy (SR) | 4 | 572-1117 | 9/2017, 4/2018, 5/2018, 7/2018, 9/2018, 4/2019, 6/2019, 8/2019, 10/2019, 6/2020, 8/2020, 10/2020 |
| Stiavnické vrchy (SR) | 3 | 481-814 | 5/2016, 5/2020, 7/2020 |
| Tríbeč (SR) | 2 | 215-420 | 5/2020, 3/2021 |
| Veľká Fatra (SR) | 6 | 451-1387 | 11/2018, 9/2019, 10/2019, 8/2020, 6/2021, 10/2021 |
| Veporské vrchy (SR) | 5 | 551-951 | 6/2019, 4/2020, 6/2020, 10/2020, 4/2021, 5/2021, 10/2021 |
| Vtáčnik (SR) | 3 | 451-1345 | 7/2020 |
| Zvolenská kotlina (SR) | 13 | 308-818 | 5/2019, 6/2019, 10/2019, 4/2020, 5/2020, 6/2020, 7/2020, 10/2020, 11/2020, 2/2021, 3/2021, 4/2021, 9/2021 |

Supplement 1 Summary of studied localities included in analyses. SK means Slovak Republic, CZ means Czech Republic. N means number of studied localities. Elevation is a range of altitudes in localities in particular orographic unit.
