# Supplementary material for: A Holistic Approach to Parasitoid–Host Interaction Along an Elevational Gradient Revealed Coevolution Driven by Host Foraging Strategy
Source: Ecol Evol. 2025 Apr 11;15(4):e71227. doi: 10.1002/ece3.71227 (PMC11991926; doi:10.1002/ece3.71227)
Supplement: Supplementary file 2 — Appendix S2 Number (N) and Parasitism rate (P) of collected spiders collected in the study. [file ECE3-15-e71227-s002.docx]

Supplement 2 Number (N) and Parasitism rate (P) of collected spiders collected in study.

| **Family** | ***Species*** | **Guild** | **N** | **P** |
| --- | --- | --- | --- | --- |
| **Araneidae** |  |  | **7204** | **0.63** |
| Araneidae | *Aculepeira ceropegia* (Walckenaer, 1802) | orbweb | 74 | 0 |
| Araneidae | *Agalenatea redii* (Scopoli, 1763) | orbweb | 2 | 0 |
| Araneidae | *Araneus alsine* (Walckenaer, 1802) | orbweb | 1 | 0 |
| Araneidae | *Araneus angulatus* Clerck, 1757 | orbweb | 13 | 0 |
| Araneidae | *Araneus diadematus* Clerck, 1757 | orbweb | 255 | 0.43 |
| Araneidae | *Araneus marmoreus* Clerck, 1757 | orbweb | 8 | 0 |
| Araneidae | *Araneus nordmanni* (Thorell, 1870) | orbweb | 28 | 0 |
| Araneidae | *Araneus quadratus* Clerck, 1757 | orbweb | 2 | 0 |
| Araneidae | *Araneus* sp. | orbweb | 357 | 0 |
| Araneidae | *Araneus sturmi* (Hahn, 1831) | orbweb | 2093 | 0.68 |
| Araneidae | *Araneus triguttatus* (Fabricius, 1775) | orbweb | 108 | 0.74 |
| Araneidae | *Araniella alpica* (L. Koch, 1869) | orbweb | 860 | 0.26 |
| Araneidae | *Araniella cucurbitina* (Clerck, 1757) | orbweb | 1383 | 0.8 |
| Araneidae | *Araniella opisthographa* (Kulczyński, 1905) | orbweb | 167 | 0.54 |
| Araneidae | *Araniella* sp. | orbweb | 959 | 1.31 |
| Araneidae | *Argiope bruennichi* (Scopoli, 1772) | orbweb | 1 | 0 |
| Araneidae | *Cyclosa conica* (Pallas, 1772) | orbweb | 200 | 0.7 |
| Araneidae | *Gibbaranea bituberculata* (Walckenaer, 1802) | orbweb | 26 | 0 |
| Araneidae | *Gibbaranea gibbosa*(Walckenaer, 1802) | orbweb | 18 | 0 |
| Araneidae | *Gibbaranea omoeda*(Thorell, 1870) | orbweb | 61 | 0.49 |
| Araneidae | *Gibbaranea* sp. | orbweb | 54 | 0.19 |
| Araneidae | *Hypsosinga heri* (Hahn, 1831) | orbweb | 2 | 0 |
| Araneidae | *Hypsosinga pygmaea* (Sundevall, 1831) | orbweb | 1 | 0 |
| Araneidae | *Hypsosinga sanguinea* (C. L. Koch, 1844) | orbweb | 25 | 0 |
| Araneidae | *Hypsosinga* sp. | orbweb | 6 | 0 |
| Araneidae | *Larinioides patagiatus* (Clerck, 1757) | orbweb | 2 | 0 |
| Araneidae | *Larinioides sclopetarius* (Clerck, 1757) | orbweb | 2 | 0 |
| Araneidae | *Larinioides* sp. | orbweb | 17 | 0 |
| Araneidae | *Mangora acalypha* (Walckenaer, 1802) | orbweb | 222 | 0 |
| Araneidae | *Nuctenea umbratica* (Clerck, 1757) | orbweb | 7 | 0 |
| Araneidae | *Singa hamata* (Clerck, 1757) | orbweb | 7 | 0 |
| Araneidae | *Singa nitidula* C. L. Koch, 1844 | orbweb | 8 | 0 |
| Araneidae | *Zilla diodia* (Walckenaer, 1802) | orbweb | 78 | 0 |
| Araneidae | *Zygiella montana* (C. L. Koch, 1834) | orbweb | 65 | 0 |
| Araneidae | *Zygiella* sp. | orbweb | 92 | 0 |
| **Dictynidae** |  |  | **2358** | **0.31** |
| Dictynidae | *Argenna subnigra* (O. P.-Cambridge, 1861) | tangleweb | 5 | 0 |
| Dictynidae | *Brigittea latens* (Fabricius, 1775) | tangleweb | 3 | 0 |
| Dictynidae | *Dictyna arundinacea* (Linnaeus, 1758) | tangleweb | 253 | 0.2 |
| Dictynidae | *Dictyna pusilla* Thorell, 1856 | tangleweb | 61 | 0 |
| Dictynidae | *Dictyna* sp. | tangleweb | 816 | 0.54 |
| Dictynidae | *Dictyna uncinata* Thorell, 1856 | tangleweb | 320 | 0.44 |
| Dictynidae | *Lathys humilis* (Blackwall, 1855) | tangleweb | 746 | 0 |
| Dictynidae | *Nigma flavescens* (Walckenaer, 1830) | tangleweb | 111 | 0.09 |
| Dictynidae | *Nigma* sp. | tangleweb | 5 | 0 |
| Dictynidae | *Nigma walckenaeri* (Roewer, 1951) | tangleweb | 38 | 2.11 |
| **Linyphiidae** |  |  | **3336** | **0.19** |
| Micronetinae | *Agnyphantes expunctus* (O. P.-Cambridge, 1875) | sheetweb | 1 | 0 |
| Micronetinae | *Agyneta affinis* (Kulczyński, 1898) | sheetweb | 5 | 0 |
| Micronetinae | *Agyneta conigera* (O. Pickard-Cambridge, 1863) | sheetweb | 46 | 0 |
| Micronetinae | *Agyneta milleri* (Thaler, Buchar & Kůrka, 1997) | sheetweb | 1 | 0 |
| Micronetinae | *Agyneta rurestris* (C. L. Koch, 1836) | sheetweb | 105 | 0 |
| Micronetinae | *Agyneta* sp. | sheetweb | 1 | 0 |
| Micronetinae | *Bathyphantes gracilis* (Blackwall, 1841) | sheetweb | 1 | 0 |
| Micronetinae | *Bathyphantes nigrinus* (Westring, 1851) | sheetweb | 1 | 0 |
| Micronetinae | *Bathyphantes* sp. | sheetweb | 15 | 0 |
| Micronetinae | *Bolyphantes alticeps* (Sundevall, 1833) | sheetweb | 1 | 0 |
| Micronetinae | *Bolyphantes luteolus* (Blackwall, 1833) | sheetweb | 1 | 0 |
| Micronetinae | *Caviphantes saxetorum* (Hull, 1916) | sheetweb | 1 | 0 |
| Micronetinae | *Centromerus dilutus* (O. P.-Cambridge, 1875) | sheetweb | 1 | 0 |
| Micronetinae | *Centromerus sylvaticus* (Blackwall, 1841) | sheetweb | 1 | 0 |
| Micronetinae | *Ceratinella brevipes* (Westring, 1851) | sheetweb | 2 | 0 |
| Micronetinae | *Diplostyla concolor* (Wider, 1834) | sheetweb | 1 | 0 |
| Micronetinae | *Drapetisca socialis* (Sundevall, 1833) | sheetweb | 6 | 0 |
| Micronetinae | *Floronia bucculenta* (Clerck, 1757) | sheetweb | 1 | 0 |
| Micronetinae | *Frontinellina frutetorum* (C. L. Koch, 1834) | sheetweb | 56 | 0 |
| Micronetinae | *Helophora insignis* (Blackwall, 1841) | sheetweb | 1 | 0 |
| Micronetinae | *Incestophantes annulatus* (Kulczyński, 1881) | sheetweb | 1 | 0 |
| Micronetinae | *Kaestneria dorsalis* (Wider, 1834) | sheetweb | 415 | 0.02 |
| Micronetinae | *Labulla thoracica*  (Wider, 1834) | sheetweb | 1 | 0 |
| Micronetinae | *Lepthyphantes* sp. | sheetweb | 24 | 0 |
| Micronetinae | *Linyphia hortensis* Sundevall, 1830 | sheetweb | 4 | 0 |
| Micronetinae | *Linyphia* sp. | sheetweb | 2 | 0 |
| Micronetinae | *Linyphia triangularis* (Clerck, 1757) | sheetweb | 112 | 0.18 |
| Micronetinae | *Megalepthyphantes collinus* (L.Koch, 1872) | sheetweb | 1 | 0 |
| Micronetinae | *Mermessus trilobatus* (Emerton, 1882) | sheetweb | 2 | 0 |
| Micronetinae | *Microlinyphia* sp. | sheetweb | 3 | 0 |
| Micronetinae | *Mughiphantes mughi* (Fickert, 1875) | sheetweb | 1083 | 0.50 |
| Micronetinae | *Neriene clathrata* (Sundevall, 1830) | sheetweb | 3 | 0 |
| Micronetinae | *Neriene emphana* (Walckenaer, 1841) | sheetweb | 12 | 0 |
| Micronetinae | *Neriene montana* (Clerck, 1757) | sheetweb | 1 | 0 |
| Micronetinae | *Neriene peltata* (Wider, 1834) | sheetweb | 771 | 0.04 |
| Micronetinae | *Neriene radiata* (Walckenaer, 1841) | sheetweb | 61 | 0.16 |
| Micronetinae | *Neriene* sp. | sheetweb | 61 | 0.33 |
| Micronetinae | *Obscuriphantes obscurus* (Blackwall, 1841) | sheetweb | 119 | 0 |
| Micronetinae | *Pityohyphantes phrygianus* (C. L. Koch, 1836) | sheetweb | 301 | 0 |
| Micronetinae | *Poeciloneta variegata* (Blackwall, 1841) | sheetweb | 80 | 0 |
| Micronetinae | *Porrhomma microphthalmum* (O. P.-Cambridge, 1871) | sheetweb | 7 | 0 |
| Micronetinae | *Porrhomma oblitum* (O. P.-Cambridge, 1871) | sheetweb | 1 | 0 |
| Micronetinae | *Porrhomma pygmaeum* (Blackwall, 1834) | sheetweb | 2 | 0 |
| Micronetinae | *Syedra gracilis* (Menge, 1869) | sheetweb | 1 | 0 |
| Micronetinae | *Tapinopa longidens* (Wider, 1834) | sheetweb | 1 | 0 |
| Micronetinae | *Tenuiphantes alacris* (Blackwall, 1853) | sheetweb | 3 | 0 |
| Micronetinae | *Tenuiphantes cristatus* (Menge, 1866) | sheetweb | 6 | 0 |
| Micronetinae | *Tenuiphantes flavipes* (Blackwall, 1854) | sheetweb | 1 | 0 |
| Micronetinae | *Tenuiphantes mengei* (Kulczyński, 1887) | sheetweb | 1 | 0 |
| Micronetinae | *Tenuiphantes* sp. | sheetweb | 2 | 0 |
| Micronetinae | *Tenuiphantes tenuis* (Blackwall, 1852) | sheetweb | 6 | 0 |
| **Tetragnathidae** |  |  | **3141** | **0.33** |
| Tetragnathidae | *Metellina mengei* (Blackwall, 1869) | orbweb | 79 | 0 |
| Tetragnathidae | *Metellina merianae* (Scopoli, 1763) | orbweb | 1 | 0 |
| Tetragnathidae | *Metellina segmentata* (Clerck, 1757) | orbweb | 396 | 0 |
| Tetragnathidae | *Metellina* sp. | orbweb | 81 | 0 |
| Tetragnathidae | *Pachygnatha degeeri* Sundevall, 1830 | orbweb | 4 | 0 |
| Tetragnathidae | *Pachygnatha listeri* Sundevall, 1830 | orbweb | 3 | 0 |
| Tetragnathidae | *Pachygnatha* sp. | orbweb | 1 | 0 |
| Tetragnathidae | *Tetragnatha montana* Simon, 1874 | orbweb | 548 | 0.05 |
| Tetragnathidae | *Tetragnatha nigrita* Lendl, 1886 | orbweb | 17 | 0 |
| Tetragnathidae | *Tetragnatha obtusa* C. L. Koch, 1837 | orbweb | 4 | 0 |
| Tetragnathidae | *Tetragnatha pinicola* L. Koch, 1870 | orbweb | 675 | 0.24 |
| Tetragnathidae | *Tetragnatha* sp. | orbweb | 1332 | 0.63 |
| **Theridiidae** |  |  | **8762** | **0.30** |
| Theridiidae | *Achaearanea* sp. | tangleweb | 4 | 0 |
| Theridiidae | *Achaeridion conigerum* (Simon, 1914) | tangleweb | 1 | 0 |
| Theridiidae | *Anelosimus vittatus* (C. L. Koch, 1836) | tangleweb | 42 | 0 |
| Theridiidae | *Asagena phalerata* (Panzer, 1801) | tangleweb | 4 | 0 |
| Theridiidae | *Cryptachaea riparia* (Blackwall, 1834) | tangleweb | 1 | 0 |
| Theridiidae | *Dipoena erythropus* (Simon, 1881) | tangleweb | 1 | 0 |
| Theridiidae | *Dipoena melanogaster* (C. L. Koch, 1837) | tangleweb | 168 | 0 |
| Theridiidae | *Dipoena* sp. | tangleweb | 2 | 0 |
| Theridiidae | *Enoplognatha latimana* Hippa & Oksala, 1982 | tangleweb | 4 | 0 |
| Theridiidae | *Enoplognatha ovata* (Clerck, 1757) | tangleweb | 450 | 0 |
| Theridiidae | *Enoplognatha* sp. | tangleweb | 43 | 0 |
| Theridiidae | *Enoplognatha thoracica* (Hahn, 1833) | tangleweb | 1 | 0 |
| Theridiidae | *Episinus angulatus* (Blackwall, 1836) | tangleweb | 5 | 0 |
| Theridiidae | *Episinus* sp. | tangleweb | 1 | 0 |
| Theridiidae | *Episinus truncatus* Latreille, 1809 | tangleweb | 1 | 0 |
| Theridiidae | *Heterotheridion nigrovariegatum* Simon, 1873 | tangleweb | 16 | 0 |
| Theridiidae | *Lasaeola tristis* (Hahn, 1833) | tangleweb | 7 | 0 |
| Theridiidae | *Neottiura bimaculata* (Linnaeus, 1767) | tangleweb | 380 | 0.53 |
| Theridiidae | *Ohlertidion ohlerti* (Thorell, 1870) | tangleweb | 113 | 0 |
| Theridiidae | *Paidiscura pallens* (Blackwall, 1834) | tangleweb | 925 | 0 |
| Theridiidae | *Parasteatoda lunata* (Clerck, 1757) | tangleweb | 18 | 0 |
| Theridiidae | *Parasteatoda simulans* (Thorell, 1875) | tangleweb | 2 | 0 |
| Theridiidae | *Parasteatoda* sp. | tangleweb | 1 | 0 |
| Theridiidae | *Phylloneta impressa* (L. Koch, 1881) | tangleweb | 380 | 0.08 |
| Theridiidae | *Phylloneta sisyphia* (Clerck, 1757) | tangleweb | 1822 | 0.07 |
| Theridiidae | *Phylloneta* sp. | tangleweb | 61 | 0.49 |
| Theridiidae | *Platnickina tincta* (Walckenaer, 1802) | tangleweb | 1528 | 0.33 |
| Theridiidae | *Robertus arundineti* (O. P.-Cambridge, 1871) | tangleweb | 3 | 0 |
| Theridiidae | *Robertus lividus* (Blackwall, 1836) | tangleweb | 5 | 0 |
| Theridiidae | *Robertus neglectus* (O. P.-Cambridge, 1871) | tangleweb | 1 | 0 |
| Theridiidae | *Robertus scoticus* Jackson, 1914 | tangleweb | 11 | 0 |
| Theridiidae | *Robertus* sp. | tangleweb | 3 | 0 |
| Theridiidae | *Rugathodes instabilis* (O. P.-Cambridge, 1871) | tangleweb | 11 | 0 |
| Theridiidae | *Simitidion simile* (C. L. Koch, 1836) | tangleweb | 15 | 0 |
| Theridiidae | *Steatoda triangulosa* (Walckenaer, 1802) | tangleweb | 1 | 0 |
| Theridiidae | *Theonoe minutissima* (O. Pickard-Cambridge, 1879) | tangleweb | 1 | 0 |
| Theridiidae | *Theridion boesenbergi* Strand, 1904 | tangleweb | 203 | 1.87 |
| Theridiidae | *Theridion melanurum* Hahn, 1831 | tangleweb | 2 | 0 |
| Theridiidae | *Theridion mystaceum* L.Koch, 1870 | tangleweb | 111 | 0.09 |
| Theridiidae | *Theridion pictum* (Walckenaer, 1802) | tangleweb | 3 | 0 |
| Theridiidae | *Theridion pinastri* L. Koch, 1872 | tangleweb | 153 | 0.26 |
| Theridiidae | *Theridion* sp. | tangleweb | 677 | 1.51 |
| Theridiidae | *Theridion varians* Hahn, 1833 | tangleweb | 1581 | 0.18 |
